# The role of COVID-19 in myopathy: incidence, causes, treatment, and prevention

**DOI:** 10.25122/jml-2022-0167

**Published:** 2022-12

**Authors:** Manal Awad, Hany Akeel Al-hussaniy, Ali Hikmat Alburghaif, Karam Turath Tawfeeq

**Affiliations:** 1 Department of Family Physician (CCFP), Madigan Medical Centre, Calgary, Alberta, Canada; 2 Department of Pharmacology, College of Medicine, University of Baghdad, Baghdad, Iraq; 3 Dr. Hany Akeel Institute, Iraqi Medical Research Center, Baghdad, Iraq; 4 Department of Pharmacy, Ashur University College, Baghdad, Iraq; 5 Department of Pathology, College of Medicine, University of Mosul, Mosul, Iraq

**Keywords:** SARS-CoV-2, COVID-19, search engine, muscular diseases, intensive care units

## Abstract

Myopathy is a disease characterized by muscle dysfunction in general and may be associated with genetics, medication such as statins, or inflammation. In 2019, an epidemic viral infection (SARS-CoV-2 virus) that invaded most countries worldwide appeared and caused acute respiratory disease. Consequently, patients had to take a group of drugs for a relatively long treatment period. According to several studies, there was an increase in the cases of muscular disorders due to several factors. This study aimed to (1) investigate the relationship between COVID-19 and myopathy and (2) identify the causes and prevention methods. A systematic review was conducted, analyzing several articles from the following databases: ResearchGate, Medline, DOAJ (The Directory of Open-Access Journals), PubMed, and Google Scholar. After performing the search and filtering the results, we included 61 articles. There was a strong relationship between COVID-19 and myopathy, especially in patients admitted to the ICU department, due to medication or neurological dysregulation with multiorgan dysfunctions such as polyneuropathy, peripheral nerve involvement, dysautonomia, Guillain-Barré syndrome, and many others.

## INTRODUCTION

Myopathies are neuromuscular diseases characterized by the disruption of the structure or development of the striated skeletal muscle, distinguishing two categories: genetic (hereditary) myopathies and acquired myopathies [1, 2]. The first data in the literature referred to hereditary myopathies (MH) in 1882, when Edward Meryon described granular degeneration in postmortem muscle [3].

Idiopathic inflammatory myopathy (IIM) directly affects skeletal muscle and can sometimes affect the skin and other organs to varying degrees. The weakness associated with critical illness is a general term that describes neuromuscular disorders associated with serious illnesses, such as COVID-19 [4, 5]. It is divided into three groups depending on the site of involvement: critical disease polyneuropathy (CIP), serious disease myopathy (CIM), and critical disease polyneuromyopathy (CIPNM)[6, 7].

Although the most common clinical manifestations caused by the SARS-CoV-2 virus described in the literature are respiratory symptoms, fever, and gastrointestinal problems, neurological symptoms such as headache, anosmia, myalgia, insomnia, and confusion were also reported [8].

Intensive care units (ICUs) represent one of the important steps in the comprehensive patient care system, and their main objective is the diagnosis and treatment of patients in critical situations, that is, with very deteriorating health conditions and a high risk of suffering severe complications in the short and medium term [9, 10].

The consequences of admission to the ICU may result from different aggressive treatments or secondary to other aspects of the same disease, such as fatigue, asthenia, weight loss, cognitive deficits, myopathy, and polyneuropathy [11, 12].

The current review aimed to investigate the relationship between myopathy and COVID-19 in critical patients hospitalized in the ICU, observing the conditions that cause several functional deteriorations in these patients [12, 13]. This is especially relevant given the current circumstances, constituting a relevant topic for future research.

## MATERIAL AND METHODS

### Search strategy

**Figure 1:**
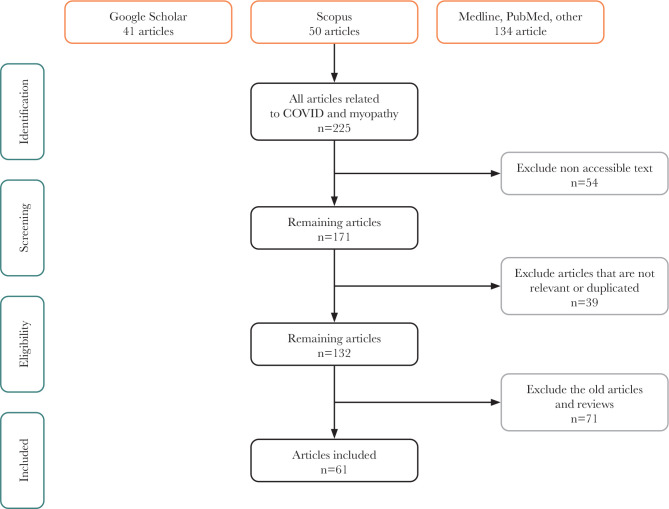
Flow diagram of the articles selection.

We conducted an unstructured search on PubMed, Google Scholar, and Redalyc using the following keywords: “COVID-19”, “myopathies”, “intensive care unit”, “critical illness”, and “consequences”. A total of 225 related articles were retrieved, and emphasis was placed on original articles, including systematic reviews, case studies, and experimental studies. We rejected 87 publications prior to 2012, resulting in 171 articles in the sampling phase, of which 39 were not relevant or duplicated, for a total of 132 to choose from. Finally, 61 of the current and best-documented publications were selected (Figure 1).

## LITERATURE REVIEW

Even though the differential diagnoses of CIM, CIP, and CIPNM are indistinguishable, there are significant differences in their pathogenesis. CIP is a sensory axonal polyneuropathy characterized by the loss of individual nerve fibers. In contrast, CIM is characterized by a decrease in thick myofilaments, which leads to the death of skeletal muscle myofibers [12].

CIP has worse results compared to CIM [12, 13]. Despite significant advances in improving knowledge of the mechanisms underlying these disorders, the period and duration of stay in the ICU are indicators of important long-term results [13, 14].

### Angiotensin-converting enzyme (ACE) and its rule in the muscle function

**Figure 2:**
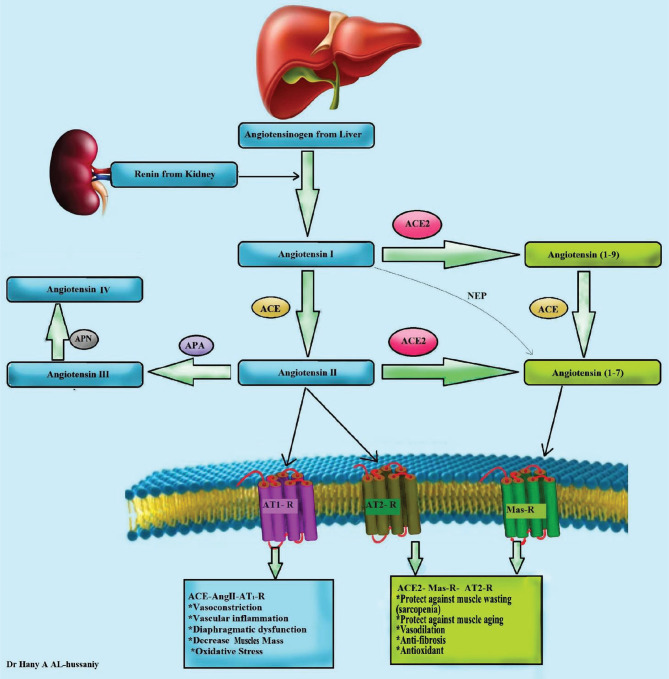
Angiotensin-converting enzyme 1,2 and its rule in COVID-19 ACE, AT-R angiotensin receptor, NEP- neprilysin, APA- Aminopeptidase A, APN- Aminopeptidase N.

One of the major hemostatic functions in the body is the renin-angiotensin aldosterone system (RAAS). The juxtaglomerular apparatus produces renin that converts angiotensinogen to angiotensin I. This angiotensin I is also affected by the enzyme angiotensin-converting enzyme (ACE) to angiotensin II converting enzyme II which either binds directly to its AT-1 receptor (and produces a harmful effect) or is converted to angiotensin 1-7 by the enzyme ACE 2 (Figure 2).

ACE2 receptors serve as a self-protective fragment, maintain biological homeostasis, and prevent the progression of numerous diseases. The interaction of SARS-Cov2 with ACE2 not only promotes virus entrance into the human body but also modifies the protective activity of ACE2 in affected organs. As a result, it is critical to reconsider the role of ACE2 in modifying organ function and human health [15].

Skeletal muscle is vital for motor system modulation as well as metabolic homeostasis. The ACE-Ang II-AT1 axis contributes to muscle pathogenesis by promoting muscle activity and glycaemic control problems, *i.e*., muscle atrophy followed by abnormal muscular remodeling or insulin resistance [15, 16].

Previous studies in striated muscles emphasize the protective effect of Ang 1-7 in pathological remodeling and that it raises blood glucose through insulin resistance [16–18]. Riquelme et al. studied the role of ACE2 in existing pathological muscle modulation, its effect and protein levels, and its variation linked to genetic factors leading to muscular dysfunction. This aspect could imply a compensatory mechanism of the RAAS front to muscular dysfunctions [19, 20].

### Pathophysiological mechanisms of muscle weakness in COVID-19

Myopathy can be caused by several factors, such as direct viral damage to skeletal muscle or secondary disorders, such as motor neuron involvement, malnutrition, the inflammatory response of CIM, prolonged bedding, and insufficient O_2_ intake [21, 22].

Atrophy and fibrosis are caused by pathological muscle remodeling of skeletal muscle, secondary to endothelial dysfunction due to CIM, the effects on systemic circulation, and the RAS system in the musculature [23].

Muscle deterioration is caused by inequity in cellular and fiber structure and metabolism, especially by suppressing the IGF-1-AKT-mTOR pathway by altering the synthesis of muscle proteins caused by Ang II, which can indirectly induce cellular apoptosis. This damaged or atrophied muscle tissue directly influenced by Ang II is replaced by connective tissue that produces muscle fibrosis with a subsequent decrease in skeletal muscle [22, 23].

In summary, it can also be mentioned that SARS-COV2 can induce muscle atrophy through regulation of the renin-angiotensin-aldosterone pathway (RAS) in interaction with other processes secondary to the hospitalization in the ICU (Figure 2) [23, 24].

### Patients with COVID-19 and myopathy

During the COVID-19 pandemic, neuromuscular complications were evident [25]. Between 30% and 50% of the ICU patients presented generalized neuromuscular weakness during their hospitalization that appears to be secondary to CIM and CIP or their combination, causing prolongation and increasing admission time and ventilation duration in the ICU [25, 26].

Montalvan et al. (2020) stated that 36.4% of patients had neurological manifestations that were directly related to the severity of the clinical symptoms of COVID-19 [26]. Santos et al. (2018) identified that patients hospitalized longer in the ICU presented physical sequelae, such as myopathy, polyneuropathy, and musculoskeletal retraction [27]. Authors such as Santos and collaborators (2021) report that the advanced phase of COVID-19 was linked to severe peripheral nerve dysfunction, dysautonomia, and myopathy [26]. Muscle weakness due to immobility after the ICU has been considered the sequelae that mostly affects these patients' quality of life and recovery time [28].

### Other causes of myopathy in COVID-19 patients

Many patients suffering from COVID-19 require admission and an increased stay in intensive care units (ICU), so the likelihood of complications and the development of CIM is highly possible [29].

### Guillain Barré syndrome (GBS) and COVID-19

GBS is among the most common causes of paralysis worldwide, manifesting acute inflammatory polyradiculoneuropathy. Common symptoms include ascending symmetric weakness, painful paranesthesia, decreased or absent osteotendinous reflexes, alteration of the cranial nerves, and in severe cases, even weakness of the respiratory muscles can occur. It can present as a heterogeneous disease, and different variants have been reported [30, 31].

Abu-Rumeileh et al. (2021) reported 73 cases with classical GBS or its variants in patients diagnosed with COVID-19, suggesting a notable increase during the pandemic [31]. Bagnato et al. assume that respiratory failure in patients with COVID-19 is secondary to the relationship between muscle weakness and viral lung infection [32].

### Transverse myelitis and encephalomyelitis in COVID-19

Transverse myelitis leads to the destruction of myelin from nerve cells, causing the interruption of the messages sent by the spinal cord throughout the body through the peripheral nerves, causing pain, muscle weakness, paralysis, sensory problems, or dysfunction of the bladder and intestine [33]. SARS-CoV-2 has clinical features that are not limited only to the respiratory tract but also compromise the nervous system. The search for indirect and direct mechanisms involved in the complications and neurological manifestations reported in COVID-19 has been the subject of studies by several authors, identifying possible mechanisms of direct invasion of SARS-CoV-2 into the CNS [34]. Thus, isolated cases of encephalopathy, encephalitis, encephalomyelitis, and hemorrhagic necrotizing encephalopathy, among others, have been described. [35, 36]

### Cerebrovascular disease (CVD) and COVID-19

Related to the prothrombotic phenotype of severe COVID, cerebrovascular events of ischemia have been reported with an incidence that can reach up to 5% of hospitalized patients [37].

Another mechanism mentioned is endothelial dysfunction; SARS-CoV-2 binds to and invades vascular endothelial cells through ACE2 in the endothelium, triggering the death of inflammatory endothelial cells (pyroptosis) [34].

### Diagnosis

CIP, CIM, and CIPNM are usually diagnosed during the recovery phase of the disease. Although they all share common clinical signs, diagnosis also depends on the correct interpretation of data related to clinical presentation and electrodiagnostic, with standard laboratory tests being insignificant [38].

Flaccidity and usually symmetrical weakness are aspects present in CIP, CIM, and CIPNM; a potential differentiation factor may be the null or decreased response to sensitivity to pain, vibration, or temperature in those with CIP, while these functions would be present in CIM [35, 39].

Muscle atrophy is generally more evident in CIM, which is difficult to diagnose due to the condition of critically ill patients. In awake patients, muscle strength can be assessed using the Medical Research Council (MRC) scale, which rates muscle weakness in six muscle groups, giving scores between 0 (no contraction) and 5 (normal force). The CIPNM is defined when the total CRF score is less than 48, having previously ruled out other causes of weakness [39, 40].

Although laboratory testing is not required to detect critical illness-associated weakness (CIAW), plasma IL-6 was already reported as an important indicator of membrane disruption in CIM [36]. GDF-15 is a stress-stimulated mediator of major muscle atrophy illnesses and is thought to be a promising candidate biomarker; however, additional research is needed to prove its utility [41, 42].

Needle electromyography can help clarify the diagnosis, early recruitment and small amplitude polyphase waves in the different affected muscles (biceps brachii muscles, rectus femoris, and anterior tibial), reduction of the amplitude and duration of the motor unit potential, and sometimes the presence of fibrillations and fasciculations.

Muscle biopsy in the case of CIP demonstrates atrophy due to the denervation of muscle fiber types 1 and 2. CIM describes myofiber atrophy, focal or diffuse loss of thick filaments, angled fibers, fat degeneration, and necrosis [43].

With imaging techniques such as computed tomography and nuclear magnetic resonance imaging, the degree of muscle loss and infiltration of muscle by adipose tissue can be observed. It also allows the evaluation of deeper muscle groups; however, some main disadvantages are high cost, radiation exposure, and the need to transport the patient out of the ICU [37, 44].

### Therapeutics

The treatment should minimize associated risk factors, symptom care, and physical rehabilitation. Some authors argue that pharmacological treatments are not recommended to prevent or treat critical illness-associated weakness (CIAW). However, euglycemia has improved some outcomes in critically ill patients. Insulin therapy greatly reduced CIP and CIM rates, including hospitalization time by mechanical ventilation and mortality rate [45].

Functional electrical stimulation (FES) has been reported beneficial in patients who have not been admitted to the ICU, promoting increased muscle strength [44]. However, the results are discordant, so more and better studies are required to support their efficacy [42].

Performing passive and active mobilization and early anesthesia breaks can aid recovery, especially in patients admitted to the intensive care unit for COVID-19 [28, 42]. It is a relatively safe procedure with a low risk of adverse events. [43] Cheung et al. (2021) report that early mobilization leads to a lower incidence of CIAW, improving functional capacity and increasing standing capacity [11]. The exercise protocols focused on transfers (from the supine to sitting), walking, and cycle ergometry adjacent to the bed [37]. Zhou et al. (2021) mention that malnutrition is the leading cause of "critical illness polyneuropathy", stressing the harmful effects of parenteral nutrition in critically ill patients and supporting early enteral feeding [39]. McGlory et al. report that omega-3 fatty acid supplementation improves skeletal muscle anabolism [40] and has potent anti-inflammatory properties [41].

## CONCLUSIONS

Myopathies are important in developing complications and sequelae associated with COVID-19 in critically ill patients admitted to the ICU. Among the most important neurological problems associated with COVID-19 and multisystem involvement are polyneuropathy, myopathy of the critical patient, and the Guillan-Barré syndrome. There is a close relationship between myopathy and COVID-19, an aspect of special importance in the current circumstances.

## References

[1] Mukund K, Subramaniam S (2020). Skeletal muscle: A review of molecular structure and function, in health and disease. Wiley Interdiscip Rev Syst Biol Med.

[2] Danielsson O, Häggqvist B (2021). Skeletal muscle immunohistochemistry of acquired and hereditary myopathies. Curr Opin Rheumatol.

[3] Dubowitz V (1998). What's in a name? Muscular dystrophy revisited. European Journal of Paediatric Neurology.

[4] Rayavarapu S, Coley W, Kinder TB, Nagaraju K (2013). Idiopathic inflammatory myopathies: pathogenic mechanisms of muscle weakness. Skelet Muscle.

[5] Al-hussaniy HA, Altalebi RR, Tylor FM, Alwash AH (2022). Leptin Hormone: In Brief. Med Pharm J.

[6] Thabet Mahmoud A, Tawfik MA, Abd El Naby SA, Abo El Fotoh WM (2018). Neurophysiological study of critical illness polyneuropathy and myopathy in mechanically ventilated children; additional aspects in paediatric critical illness comorbidities. European Journal of Neurology.

[7] Shepherd S, Batra A, Lerner DP (2017). Review of critical illness myopathy and neuropathy. Neurohospitalist.

[8] Tankisi H, de Carvalho M, Z'Graggen WJ (2020). Critical illness neuropathy. Journal of Clinical Neurophysiology.

[9] Intiso D, Centra AM, Giordano A, Santamato A (2022). Critical illness polyneuropathy and functional outcome in subjects with COVID-19: Report on four patients and a scoping review of the literature. J Rehabil Med.

[10] Abdulameer AA, Mohammed ZN, Tawfeeq KT (2022). Endoscopic characteristics and management of Subepithelial Lesions in Video-Gastascopie. Med Pharm J.

[11] Cheung K, Rathbone A, Melanson M, Trier J (2021). Pathophysiology and management of critical illness polyneuropathy and myopathy. Journal of Applied Physiology.

[12] Versace V, Sebastianelli L, Ferrazzoli D, Saltuari L (2021). Case report: Myopathy in critically ill COVID-19 patients: A consequence of hyperinflammation?. Front Neurol.

[13] Naji MA, Alburghaif AH, Saleh NK (2022). Patient expectations regarding consultation with a family doctor: a cross-sectional study. Med Pharm J.

[14] Villa D, Ardolino G, Borellini L, Cogiamanian F (2021). Subclinical myopathic changes in COVID-19. Neurological Sciences.

[15] ALZobaidy MA, AlbuRghaif AH, Alhasany HA, Naji MA (2021). Angiotensin-converting enzyme inhibitors may increase the risk of severe COVID-19 infection. Annals of the Romanian Society for Cell Biology.

[16] Al-Kuraishy HM, Al-Gareeb AI, Al-Hussaniy HA, Al-Harcan NAH, Alexiou A, Batiha GE (2022). Neutrophil Extracellular Traps (NETs) and COVID-19: A new frontiers for therapeutic modality. Int Immunopharmacol.

[17] Vrettou CS, Mantziou V, Vassiliou AG, Orfanos SE (2022). Post-intensive care syndrome in survivors from critical illness including COVID-19 patients: A narrative review. Life (Basel).

[18] Ercegovac M, Asanin M, Savic-Radojevic A, Ranin J (2022). Antioxidant Genetic Profile Modifies Probability of Developing Neurological Sequelae in Long-COVID. Antioxidants.

[19] Riquelme C, Acuña MJ, Torrejón J, Rebolledo D (2014). ACE2 is augmented in dystrophic skeletal muscle and plays a role in decreasing associated fibrosis. PloS one.

[20] Seixas MLGA, Mitre LP, Shams S, Lanzuolo GB (2022). Unraveling muscle impairment associated with COVID-19 and the role of 3D culture in its investigation. Front Nutr.

[21] Gonzalez A, Orozco-Aguilar J, Achiardi O, Simon F, Cabello-Verrugio C (2020). SARS-CoV-2/Renin-Angiotensin System: Deciphering the Clues for a Couple with Potentially Harmful Effects on Skeletal Muscle. Int J Mol Sci.

[22] Yoshida T, Delafontaine P (2020). Mechanisms of IGF-1-Mediated Regulation of Skeletal Muscle Hypertrophy and Atrophy. Cells.

[23] Cabello-Verrugio C, Córdova G, Salas JD (2012). Angiotensin II: role in skeletal muscle atrophy. Curr Protein Pept Sci.

[24] Kingsley J, Torimoto K, Hashimoto T, Eguchi S (2021). Angiotensin II inhibition: a potential treatment to slow the progression of sarcopenia. Clin Sci (Lond).

[25] Al-hassany HA, Albu-rghaif AH A, Naji M (2021). Tumor diagnosis by genetic markers protein P-53, p16, C-MYC, N-MYC, protein K-Ras, and gene her-2 Neu is this possible?. Pakistan Journal of Medical and Health Sciences.

[26] Montalvan V, Lee J, Bueso T, De Toledo J, Rivas K (2020). Neurological manifestations of COVID-19 and other coronavirus infections: A systematic review. Clin Neurol Neurosurg.

[27] Santos RAS, Sampaio WO, Alzamora AC, Motta-Santos D (2018). The ACE2/Angiotensin-(1-7)/MAS Axis of the Renin-Angiotensin System: Focus on Angiotensin-(1-7). Physiol Rev.

[28] Al-hussaniy HA, Altalebi RR, Alburagheef A, Abdul-Amir AG (2022). The Use of PCR for Respiratory Virus Detection on the Diagnosis and Treatment Decision of Respiratory Tract Infections in Iraq. J Pure Appl Microbiol.

[29] McWilliams D, Weblin J, Hodson J, Veenith T (2021). Rehabilitation levels in patients with COVID-19 admitted to intensive care requiring invasive ventilation. An observational study. Ann Am Thorac Soc.

[30] Al-Juhaishi AM, Aziz ND (2022). Safety and Efficacy of antiviral drugs against COVID19 infection: an updated systemic review. Medical and Pharmaceutical Journal.

[31] Abu-Rumeileh S, Abdelhak A, Foschi M, Tumani H, Otto M (2021). Guillain-Barré syndrome spectrum associated with COVID-19: an up-to-date systematic review of 73 cases. J Neurol.

[32] Bagnato S, Boccagni C, Marino G, Prestandrea C (2020). Critical illness myopathy after COVID-19. International Journal of Infectious Diseases.

[33] Al-hussaniy HA, Al-tameemi ZS (2022). Methicillin-Resistant Staphylococcus aureus and New Delhi Metallo beta-lactamases-types of antibiotic resistance, methods of prevention. Med Pharm J.

[34] Haidar MA, Shakkour Z, Reslan MA, Al-Haj N (2022). SARS-CoV-2 involvement in central nervous system tissue damage. Neural Regen Res.

[35] Cabañes-Martínez L, Villadóniga M, González-Rodríguez L, Araque L (2020). Neuromuscular involvement in COVID-19 critically ill patients. Clin Neurophysiol.

[36] Al-kuraishy AA, Jalil HJ, Mahdi AS, Al-hussaniy HA (2022). General anesthesia in patient with Brain Injury. Med. Pharm. J.

[37] Dietmann A, Ripellino P, Humm AM, Hundsberger T (2022). Hot Topics on COVID-19 and Its Possible Association with Guillain-Barré Syndrome. Clinical and Translational Neuroscience.

[38] Feier CVI, Muntean C, Bardan R, Olariu A, Olariu S (2022). Impact of COVID-19 pandemic on a general surgery clinic. J Med Life.

[39] Zhou W, Ruksakulpiwat S, Fan Y, Ji L (2021). Nutritional Interventions on Physical Functioning for Critically Ill Patients: An Integrative Review. J Multidiscip Healthc.

[40] McGlory C, Calder PC, Nunes EA (2019). The Influence of Omega-3 Fatty Acids on Skeletal Muscle Protein Turnover in Health, Disuse, and Disease. Front Nutr.

[41] Sawada Y, Saito-Sasaki N, Nakamura M (2021). Omega 3 Fatty Acid and Skin Diseases. Front Immunol.

[42] Akeel Naji H (2021). The Psychosocial and Economic Impact of Uveitis in Iraq. RABMS.

[43] Antonescu F, Adam M, Popa C, Tuţă S (2018). A review of cervical spine MRI in ALS patients. J Med Life.

[44] Parry SM, Berney S, Koopman R, Bryant A (2012). Early rehabilitation in critical care (eRiCC): functional electrical stimulation with cycling protocol for a randomised controlled trial. BMJ open.

[45] Al-Hussaniy HA, Alburghaif AH, Naji MA (2021). Leptin hormone and its effectiveness in reproduction, metabolism, immunity, diabetes, hopes and ambitions. J Med Life.

